# Attention Guided Feature Encoding for Scene Text Recognition

**DOI:** 10.3390/jimaging8100276

**Published:** 2022-10-08

**Authors:** Ehtesham Hassan, Lekshmi V. L.

**Affiliations:** Department of Computer Science and Engineering, Kuwait College of Science and Technology, Doha District, Block 4, Kuwait City 35004, Kuwait

**Keywords:** scene text recognition, convolutional neural network, LSTM, recurrent neural network

## Abstract

The real-life scene images exhibit a range of variations in text appearances, including complex shapes, variations in sizes, and fancy font properties. Consequently, text recognition from scene images remains a challenging problem in computer vision research. We present a scene text recognition methodology by designing a novel feature-enhanced convolutional recurrent neural network architecture. Our work addresses scene text recognition as well as sequence-to-sequence modeling, where a novel deep encoder–decoder network is proposed. The encoder in the proposed network is designed around a hierarchy of convolutional blocks enabled with spatial attention blocks, followed by bidirectional long short-term memory layers. In contrast to existing methods for scene text recognition, which incorporate temporal attention on the decoder side of the entire architecture, our convolutional architecture incorporates novel spatial attention design to guide feature extraction onto textual details in scene text images. The experiments and analysis demonstrate that our approach learns robust text-specific feature sequences for input images, as the convolution architecture designed for feature extraction is tuned to capture a broader spatial text context. With extensive experiments on ICDAR2013, ICDAR2015, IIIT5K and SVT datasets, the paper demonstrates an improvement over many important state-of-the-art methods.

## 1. Introduction

Text appearances in natural scenes exhibit much larger variations in fonts, scripts, and scale, including curved and complex shapes, unlike conventional scanned document images. In addition, text appearances in scenes are incidental in nature. The recognition of text contents in scene images requires a robust feature extraction approach to capture the text appearances in all forms and the encoding of extracted features in a sequential form for subsequent analysis to assign character labels. The unique nature of the problem requires an alternate strategy, unlike optical character recognition techniques. With the advancements in deep learning research, many recent works on scene text recognition have applied deep-neural-network-based methods to solve this problem [1,2,3]. However, despite significant progress, scene text recognition remains a challenging task in computer vision research due to the increasing variations in text appearances observed in their curved shapes, arbitrary orientations, size variations, and fancy font styles, etc.

Our work presents a novel deep neural network for recognizing text segments in natural scene images, which applies spatial attention-enabled convolutional architecture to feature extraction. The features are subsequently processed using LSTM recurrent neural network (RNN) layers to generate text transcriptions. Our approach addresses the image to text label generation as conventional sequence-to-sequence mapping. The connectionist temporal classification (CTC) [2] and neural translation [4] are well-known sequence-to-sequence mapping methods, which build upon RNN-based encoder–decoder formulations to learn the sequence alignment between input features and labels. The proposed network is designed along the same lines: the encoder consists of a novel convolutional recurrent neural architecture integrated with spatial attention blocks. This enables the encoder to generate robust feature sequences for the input image by analyzing text attributes at multiple scales. The evaluation of the proposed architecture demonstrates that our novel spatial attention block design can significantly enhance the feature extraction process in a simple convolutional neural architecture. The major contributions of this paper are as follows:

- A novel deep neural network for scene text recognition based on an RNN-based encoder–decoder. The encoder consists of: (i) a convolutional neural network enabled with an attention mechanism to extract deep convolutional features, and (ii) bidirectional LSTM layers to convert input features into sequence representation. The VGG16 architecture is used as the basis, and is redesigned for the convolutional neural structure in the proposed method. The decoder is made up of a hierarchy of LSTM layers, and the entire proposed network is trained end-to-end, with CTC loss minimization as the learning goal. Our method demonstrates that spatial-attention-based feature extraction improves the efficacy of feature sequence encoding.
- The proposed design was thoroughly validated using ICDAR2013, ICDAR2015, IIIT5K, and SVT text datasets with a variety of geometric properties and shapes. The results from different experiments demonstrate that the proposed network is an efficient solution for recognizing natural scene segments with fancy, oriented, and curved text appearances. Further, the results also establish that the proposed method outperforms many recent methods of scene text recognition.

The structure of the paper is as follows: Section 2 presents a survey of prominent methods for natural scene text recognition. The proposed network architecture, with all relevant details, is discussed in Section 3. The experimental evaluation of the presented methods is discussed in Section 4. Section 5 concludes the paper and provides directions for future work.

## 2. Literature Survey

The recognition of text segments in scene images involves the transcription of detected segments to text labels. The earlier works in this direction focused on capturing the structural properties of character segments and processing them further for sequence recognition. The seminal work by Neumann and Matas [5] used a combination of Adaboost and a decision tree for recognition of detected extremal regions. Mishra et al. [6] proposed random field-based modeling of image features for text recognition. Strokelets discussed in [7] applied random forests for the recognition of detected strokelets. In [8], a combination of structural feature descriptors was applied for character recognition in an SVM-based model. In [9], the authors proposed discriminative feature learning for character images, exploiting the informative regions in input images.

The early deep learning methods for scene text recognition explored different ways of using convolution neural networks in the recognition task [10,11,12,13]. The PhotoOCR application in [12] demonstrated the use of deep neural networks without convolutional operations for character recognition with raw and edge-based feature representations. The recent work by Cai et al. [14] explored the image classification methodology used for scene text recognition using convolutional neural architecture. The authors in [15], demonstrated an early application of recurrent neural networks for modeling scene text using orientation features. The recent deep-learning-based methods of text segments’ recognition in scenes adopted the sequence-to-sequence modeling approach, applying recurrent neural networks [16,17,18,19]. The primary motivation comes from the fact that recurrent neural networks are naturally structured to capture the temporal context of sequential data streams. Self-attention mechanisms in neural architecture present an alternate approach to extract global dependencies between input and output streams [20]. Many existing scene text recognition methods exploit the decoder side application of temporal attention to learn the alignment between decoder hidden states and character labels [1,16,17,21]. Nevertheless, the efficacy of temporal attention depends upon the size of the available lexicon. Furthermore, the training of recurrent neural networks is a challenging task due to the vanishing and exploding gradient problems. To address this issue, many recent methods have also demonstrated the use of convolutional neural architecture in encoder–decoder formulations for scene text recognition [22,23]. In [24], Yin et al. proposed a character-level convolutional network architecture trained with CTC loss for scene text recognition. Xie et al. [23] presented a convolutional neural-network-based encoder–decoder formulation for scene text recognition incorporated with spatial and temporal attention mechanisms. In [25], the authors presented a convolutional network for incorporating temporal attention in the decoder formulation. However, convolutional networks are natural choices for modeling tasks dealing with fixed input and output dimensions, unlike the case of sequence-to-sequence modeling. In this context, Yan et al. [26] demonstrated graph convolutional neural networks for text primitive representation learning using feature aggregation at multiple scales. There are also end-to-end methods that propose a single deep neural network architecture for the detection and recognition of text segments from scene images [27,28,29,30,31]. Despite significant progress, the existing scene text recognition methods are insufficient, due to performance requirements and complex design steps. Further, the majority of existing methods heavily depend on the effectiveness of the attention mechanism in the decoding stage. We studied the scene text recognition problem, focusing on generating a robust feature sequence encoding addressing the possible variations in the input. The proposed convolutional recurrent neural-network-based encoder formulation consists of learnable spatial attention blocks. The custom-made attention blocks enable the feature sequence learning to capture textual details at multiple scales.

## 3. Convolutional Recurrent Neural Network (CRNN) for Encoder–Decoder

**Figure 1:**
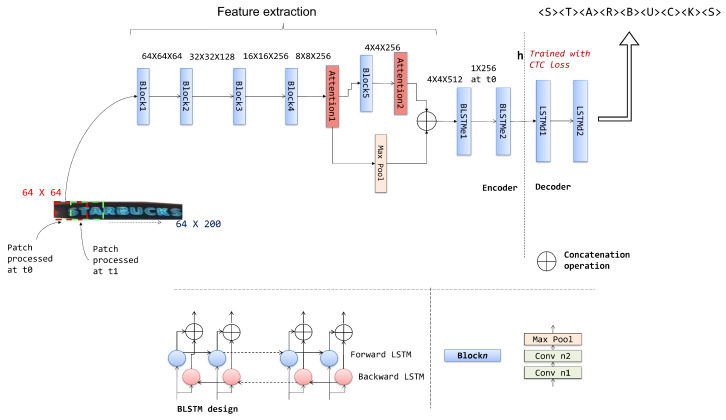
Convolutional recurrent neural architecture for scene text analysis: the bidirectional LSTM, and the convolutional block structure are given below. The dimension of intermediate feature maps is given above the connections.

The recognition of text segments in the scenes requires the transcription of image content to text labels. This requires the image modeling method to learn an efficient means of mapping between image attributes and character sequences. Many recent methods, as discussed in Section 2, focused on a convolutional neural-network-based analysis of image segments to learn the corresponding text mapping using the CTC loss training objective. Our approach follows a similar methodology, with a novel convolutional recurrent neural architecture for image content encoding using spatial attention blocks. We apply a bilinear LSTM-based encoder–decoder architecture to learn the mapping between deep convolutional feature sequences and the corresponding text labels. Figure 1 shows the architecture of the proposed scene text recognition method.

The network receives an image input of 64×200 pixels. First, the input is processed through a convolutional neural structure to extract higher-order deep convolutional features. Following that, the features are applied to a bilinear LSTM network to convert the input to a deep feature sequence representation h. The text transcription for the corresponding feature sequence h is generated by an LSTM-network-based decoder with the CTC layer to output text labels.

### 3.1. The Encoder Design

The encoder in the proposed deep neural network transforms the input image to a feature sequence representation. This requires: (i) the extraction of low-level features from the input; (ii) the conversion of features to sequence. Based on the reputation of convolutional neural architectures in feature extraction tasks, we designed the feature extraction branch using the VGG16 architecture [32] as the base. Figure 1 shows the details of the encoder network. We removed the last two fully connected layers from the original VGG16 architecture and preserved the hierarchy of five convolutional blocks. Each convolutional block Blockn consists of two convolutional layers followed by a max pool layer. The convolutional blocks exploit the patterns and textures in the input image. Our objective is to obtain a computationally efficient feature extraction structure able to capture low-level details in input. Furthermore, to impose nonlinearity in features, the convolutional layers apply the relu activation function. The output tensor abstracts the low-level details at multiple levels along the depth of the tensor towards the higher order of convolutional blocks in the network. We incorporated two attention blocks in the network, denoted as Attention1 and Attention2, to direct the feature extraction on spatial details (details discussed later). The attention block parameters are trained to emphasize text-specific discriminative features in the network learning process. Furthermore, we combined the deep features tapped from multiple levels in the network, which were subsequently encoded in a sequence using bidirectional LSTM layers.

The encoder scans the input image from left to right, and for each time stamp, a rectangular patch of 64×64 from the input is processed through the convolutional recurrent neural structure. The stride parameter of 3 pixels is used between two patches. For the given input, the bidirectional LSTM layer outputs the vector $h=(h_{1},h_{2},\cdots ,h_{T})$ where $h_{i}$ corresponds to the BLSTMe2 hidden state update after processing the *i*th path, and *T* corresponds to the number of patches in the input. The bidirectional LSTM structure consisting of a pair of LSTM layers is shown below in Figure 1. The bidirectional formulation accurately captures temporal context derived from spatial features. Here, $h_{i}=\left[h_{i}^{f};h_{i}^{b}\right]$, where $h_{i}^{f}$ and $h_{i}^{b}$ represent the forward and backward LSTM hidden state updates. Unlike existing methods, which update the encoder states by scanning the image feature map, the proposed encoder accounts for broader local text context during state updates, supported by patch-level input scanning.

### 3.2. Design of the Attention Block

**Figure 2:**
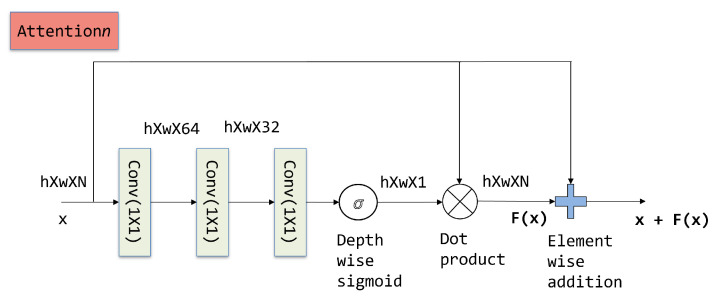
Convolutional structure for the proposed attention block. The dimension of layer outputs is mentioned above the connections.

The attention blocks incorporated in the text recognizer shown in Figure 1 emphasize text attributes in the convolutional block output tensors. This requires the attention block to capture the interdependencies between the feature channels and spatial locations. In addition, the attention block design should be computationally efficient. We designed the attention block to aggregate and separate the discriminative features in the input tensor using a hierarchy of convolutional layers with a 1×1 filter and a residual connection. Figure 2 shows the structure of the attention block design. The convolutional branch performs the aggregation of depth-wise features with dimension reduction. The output of the first two convolutional layers is passed through relu activation before being applied to the subsequent layer. The output of the third convolutional layer is gated through the depthwise sigmoid function to generate a filtered feature map F(x) for the input x. A residual connection is incorporated into the design for the efficient learning of the attention block parameters, which, when combined with convolutional branch output, results in the attention block output of F(x)+x where F can be canonically represented as

(1) F(x)=x⊗(σ(Conv(Conv(Conv(x,1×1),1×1),1×1)))

In the expression given above, ⊗ represents the spatial dot product between x and the attention map generated from the convolutional branch. σ refers to the sigmoid operation on the depth of a spatial feature location; the operation results in the attention map for input x. The dot product generates the text specific filtered feature map, which is used to amplify the text specific spatial locations in the input feature representation. The structure of our attention block is similar to the residual convolutional block used in [33]. Nevertheless, the convolutional layer output in the proposed attention block is used to learn a text specific feature filter. Further, the proposed recognition network trains on the fusion of attention block outputs extracted at different stages, as shown in Figure 1.

### 3.3. The Decoder Design

As illustrated in Figure 1, the decoder architecture for transcribing text labels from the state vector h consists of two LSTM layers. The Connectionist temporal classification (CTC) decoder proposed by Graves et al. [2] is followed to generate a text label for the given input. The encoding vector $h_{i}$ is processed through the LSTM layers at each time stamp *t*, and the LSTMd2 layer with softmax activation outputs a probability distribution over the symbol set and the most probable character label. The symbol set *L* includes all symbols under consideration, including the blank symbol.

The CTC approach models a many-to-one mapping function *B* between the probability sequences π from the LSTMd2 output, i.e., a sequence of probabilities of observing a specific label from *L* at a given time stamp *t* onto a predicted sequence, i.e., a sequence of labels of a length less than or equal to the input sequence. The mapping *B*, therefore, removes the repeated labels and the blank predictions. If $p_{k}^{t}$ represents the probability for *k* character symbol at time *t*, then the conditional probability distribution for the input sequence h over the symbol set is defined as

(2) $$p\left(\pi \;|\;h\right)=\prod_{t=1}^{T}p_{\pi_{t}}^{t}\;,\forall \pi \in L^{T}$$

The right side of Equation (2) gives the probability of single-label alignment (also referred to as path) for the input sequence h. The set $L^{T}$ corresponds to all possible text labelings of length *T*. The conditional probability of having a label sequence $\mathrm{label}\in L^{\leq T}$ for input h is the sum of probabilities of the paths π for which B(π)=label:

(3) $$p\left(\mathrm{label}\;|\;h\right)=\sum_{\pi \in B^{-\mathrm{label}}}p\left(\pi \;|\;h\right)$$

Using the conditional probability distribution, the text label for input h is computed as

(4) $${\mathrm{label}}^{*}={\mathrm{argmax}}_{L^{\leq T}}\;p\left(\mathrm{label}\;|\;h\right)$$

To compute the above expression, we adopt the best path encoding, as suggested in [2], which computes ${\mathrm{label}}^{*}$ as the concatenation of most probable output at each time stamp, assuming the most probable path corresponds to the most probable mapping, i.e., ${\mathrm{label}}^{*}\approx B\left({\mathrm{argmax}}_{\pi }p\left(\pi \;|\;h\right)\right)$. The network is trained with the objective to maximize the likelihood of the ground truth label. For the given image with corresponding encoder representation h, the training loss is calculated as

(5) $${\mathrm{ctc}}_{\mathrm{loss}}=-\log p\left(\mathrm{label}\;|\;h\right)$$

In lexicon-guided recognition, the search space in Equation (4) is restricted to the available lexicon. In this case, the equation is modified as

(6) $${\mathrm{label}}^{*}={\mathrm{argmax}}_{\mathrm{label}\in D}\;p\left(\mathrm{label}\;|\;h\right)$$

The size of the search space |D| can be minimized by limiting the search to the local neighborhood of network prediction label bounded by the maximum edit distance. In the present work, we used the BK tree [34] data structure to accelerate the search in the lexicon.

## 4. Experiments and Analysis

The methods proposed in this study are evaluated on the following datasets.

1. ICDAR2013 [35]: The dataset is a collection of natural images with horizontal and near-horizontal text appearances. The collection consists of 229 training and 233 testing images with character and word level bounding box annotations and corresponding annotations.
2. ICDAR2015 [36]: The dataset is released as the fourth challenge in the ICDAR 2015 robust reading competition (incidental scene text detection). The dataset consists of 1500 images, of which were used 1000 for training purposes and the remaining images were used for testing. The images are real-life scenes captured from Google Glass in an incidental manner, with the annotations available as quadrangle text bounding boxes with corresponding Unicode transcription.
3. IIIT5K [6]: The dataset contains a set of 3000 test and 2000 train images collected from the web. The images are associated with a short 50-word lexicon and a long 1000-word lexicon. The lexicons contain the exact ground truth word and some randomly selected words.
4. Street-view text (SVT) [28]: The dataset consists of 100 training and 250 testing images gathered from Google street view. In total, the training and testing sets consist of 211 and 514 word images. The images have an annotated axis aligned bounding-boxes around word occurrences, with corresponding labels. In addition, the images are annotated with the 50-word lexicon.

In the above-mentioned datasets, the ICDAR2015 consists of irregular images, whereas the other datasets are regular datasets.

### 4.1. Network Training and Hyperparameters

The network architecture presented in Section 1 from scratch using the Adam optimizer [37,38] with L2 regularization. Table 1 shows the hyper-parameters used for network training on different datasets, which were set experimentally following the protocols suggested in [38]. For the ICDAR2013 and SVT datasets, five fold cross-validation was applied for parameter tuning due to the small training set. The LSTM layers on the encoder side and the bidirectional LSTMs were designed with 256 hidden units. The parameter was selected to achieve the text recognition objective without increasing the training complexity and compromising on discriminability.

**Training**: We trained the proposed architecture following two different procedures:

(i) In the first type of network learning, the proposed scene text recognition network was pre-trained on a small set of examples from ICDAR2015, IIITK and SVT datasets, and then the model was trained on different evaluation datasets. The pre-training set consisted of 5% of the randomly selected training images from both datasets. The pre-training was performed for 20 epochs with a slow learning rate of 0.0001 and the batch size was fixed at 16. It was necessary to initialize the network weight parameters with the domain data distribution. The pre-training step also helps the network train on small datasets, as training from scratch on these datasets with randomly initialized weights would not be effective. For subsequent evaluations on different datasets, we tuned the network learning rate between (0.0001 and 0.005). The final learning rate and the batch size for all experiments are given in Table 1. Further, the network learning rate was reduced by half every 5 epochs after crossing half of the total number of training epochs. The images with a height bigger than width were rotated clockwise by $90^{\circ }$.
(ii) In the second type, the proposed network was trained on the Synth90k synthetic dataset [39] with an initial learning rate of 0.002 and batch size of 16. The Synth90k dataset consisted of 9 million synthetic word images generated with a dictionary of 90k English words by applying random transformations and backgrounds to word images. Each image was annotated with the corresponding word label. The network was trained for 40 epochs, with learning rate decay fixed to half after 20 epochs at the step of the 5 epochs. Again, these parameters were selected based on the discussions in [38].

### 4.2. Results and Discussion

The data in Table 2 show the recognition accuracy achieved on test datasets by applying the proposed method, following both the training procedures mentioned in Section 4.1, and the best results reported by other prominent recent methods. The datasets ICDAR2013 and ICDAR2015 do not include a lexicon. The result in bold refers to the best result. The table also shows the subsequent best four results, as underlined. As observed, the proposed method performs in the top five in many experiments with the ICDAR2015, IIIT5K, and SVT datasets. Further, on the ICDAR2013 and IIIT5K datasets, the proposed method improves on important state-of-the-arts, including RARE [19], CRNN [27], SqueezeText [17], STAR-Net [33] and RNTR-Net [40]. Our method achieves less than the TextScanner [41] in the overall comparison. Simultaneously, ESIR [30], ScRN [42] and SAR [43] perform better on IIIT5Kdataset. On the ICDAR2015 dataset, which consists of irregular text appearances, the proposed method outperformed ESIR [30], ScRN [42], AON [44], Bai et al. [16] and SAR [43]. Considering the performance of our model, which was trained following the first training procedure, it is noteworthy that we achieved a comparable performance to many state-of-the-art models by initializing the network weights on a small collection of example images, unlike other methods [16,17,25,44], which train on much larger synthetic datasets (Synth90k and SynthText). For example, TextScanner [41] uses synthetic data for pre-training, followed by tuning on evaluation datasets. Further, ESIR [30], ScRN [42] and RARE [19] employ methods to address the rectification of input images in convolutional neural architecture. Unlike the state-of-the-art, our method presents a simpler convolutional recurrent neural network architecture for scene text recognition. We observe that, in general, the proposed network trained on the Synth90K dataset performs slightly better than the model trained directly on the evaluation datasets. Nevertheless, for the ICDAR2015 dataset, the model trained on the training set achieves a recognition accuracy close to that of the model trained on the Synth90K. The dataset consists of challenging irregular images with arbitrary variations, where the state-of-the-art falls behind the other evaluation datasets. The first model, adapted to the task images, is equally effective compared to the model trained on the Synth90K dataset. The results in Table 2 establish that, despite its simple design, the proposed method can achieve a comparable or better performance than many recent methods. The proposed network focuses on more robust feature encoding for text transcription, unlike other methods, which rely on attention mechanisms on the decoder side for accurate recognition.

**Runtime Performance**: With both the attention blocks in place, the sub-optimal implementation of the proposed architecture took, on average, 0.160 seconds for text recognition in the input image. A NVIDIA Quadro P5000 GPU workstation with 32GB RAM was used for the implementation and evaluation of the proposed method.

### 4.3. Analysis of Attention Block Performance

As part of this ablation study, we evaluate the individual contribution of attention blocks in the proposed architecture. Therefore, we individually integrate Attention1 and Attention2 blocks in the network shown in Figure 1. As a result, the dimension of the input tensor to the bidirectional BLSTMe1 changes. Table 3 shows a summary of experiments, along with the average processing time in seconds. We used the Synth90K dataset to train the models used for this analysis. The experiment focused on the ICDAR2015, IIIT5K, and SVT datasets. The results demonstrate the stepwise impact of incorporating attention blocks in the architecture. It should be noted that, with a single spatial block in the proposed text recognition architecture, our method performs better than ESIR [30], AON [44] and Bai et al. [16] on the ICDAR2015 dataset. The positioning of the attention blocks is almost equally effective, as observed by the increase in recognition accuracy. However, the learning-based fusion of attention blocks’ output significantly increases the overall recognition accuracy, as observed in the final results given in Table 2. Table 4 illustrates sample images and their corresponding recognition with different configurations of attention blocks in the proposed method. The recognized labels are network output without the use of the available lexicon. The observation of text labels again establishes the results presented in Table 3. We observe that, for difficult cases of curved, multiline, and irregular text instances, such as the example shown in the fifth row of Table 4, the proposed method lacks the ability to differentiate the local features at different scales. To address such cases, the incorporation of a rectification module into the proposed architecture is a possible further direction of exploration. The incorporation of additional attention blocks in the proposed architecture at the beginning of the feature extraction stage can also be experimented with. Both options, however, would raise the overall computational cost of the recognition.

## 5. Conclusions

We presented a novel text recognition method for text segments detected from scene images. We demonstrated a novel CRNN that uses a spatio-temporal context to exploit scene text images using a spatial-attention-blocks-enabled convolutional neural network combined with LSTM-RNN layers. Unlike the recent methods, which build on residual networks to learn the image feature map, our method utilizes a novel design of spatial attention blocks integrated into a convolutional recurrent neural architecture. Further, the proposed network applies CTC and attention mechanisms to generate text labels from the given input image. With experiments on different challenging datasets, the results and analysis establish the merits of the proposed method. The incorporation of a text rectification method to address the complex cases of irregular text appearances is an important direction of future work on the proposed method.

## Figures and Tables

**Table 1:** Hyper-parameters for network training on evaluation datasets: lr represents the learning rate.

Dataset	Initial lr	# of Epochs	Batch Size	# of Epochs for lr Decay
ICDAR2013	0.001	50	16	25
ICDAR2015	0.0005	50	16	25
IIIT5K	0.0005	50	16	25
SVT	0.001	60	24	30

**Table 2:** The evaluation of the proposed text recognition method and results reported by other methods for comparison. Multiple columns corresponding to the dataset represent evaluation with different lexicons; the lexicon size is mentioned in the next row. The SynthText dataset [45] consists of 8K natural images with 8 million synthetic word instances, placed using different settings. Each text instance is annotated with a corresponding word label, and ground-truth character and word-bounding boxes.

Method	Training Data	ICDAR2013	ICDAR2015	IIIT5K			SVT	
		None	None	50	1K	None	50	None
SqueezeText * [17]	-	92.9	-	97.0	94.1	87.0	95.2	-
RARE [19]	Synth90k	88.6	-	96.2	93.8	81.9	95.5	81.9
CRNN [27]	Synth90k	86.7	-	97.6	94.4	78.2	96.4	80.8
Yin et al. [24]	Synth90k	85.2	-	98.9	96.7	81.6	95.1	76.5
STAR-Net [33]	Synth90k	89.1	-	97.7	94.5	83.3	95.5	83.6
RNTR-Net [40]	Synth90k	90.1	-	98.7	96.4	84.7	95.7	80.0
Fang et al. [21]	Synth90k	93.5	71.2	98.5	96.8	86.7	97.8	86.7
SCAN [25]	Synth90k	90.4	-	99.1	97.2	84.9	95.7	85.0
CA-FCN [22]	SynthText	91.5	-	**99.8**	98.8	91.9	98.8	86.4
ESIR [30]	Synth90k and SynthText	91.3	76.9	99.6	98.8	93.3	97.4	90.2
AON [44]	Synth90k and SynthText	-	68.2	99.6	98.1	87.0	96.0	82.8
Bai et al. [16]	Synth90k and SynthText	94.4	73.9	99.5	97.9	88.3	96.6	87.5
FAN [18]	Synth90k and SynthText	93.3	**85.3**	99.3	97.5	87.4	97.1	85.9
ScRN [42]	Synth90k and SynthText	93.9	78.7	99.5	98.8	94.4	97.2	88.9
SAR [43]	Synth90k, SynthText, real data	94.0	78.8	99.4	98.2	95.0	98.5	91.2
TextScanner [41]	Synth90k, SynthText, real data	**94.9**	83.5	**99.8**	**99.5**	**95.7**	**99.4**	**92.7**
**Ours**	real data	93.4	79.1	98.7	98.0	92.3	95.7	87.9
**Ours**	Synth90k	93.7	79.3	98.9	97.8	92.4	96.1	88.1

* synthetic data with 1 million scene text images.

**Table 3:** Analysis of attention blocks in the proposed text recognition network in different configurations.

Method	ICDAR2015	IIIT5K			SVT		Average Processing Time in Seconds
	None	50	1K	None	50	None	
Without Attention1 and Attention2	76.3	97.5	96.2	90.8	92.6	85.5	0.131
With Attention1	78.4	98.1	97.4	91.6	93.7	87.2	0.146
With Attention2	78.7	98.4	97.2	91.9	94.0	87.1	0.147

**Table 4:** Example images and recognized text labels by the proposed network under different attention block configuations.

Example Image	Without Attention	With Attention1	With Attention2	With Attention1 & Attention2
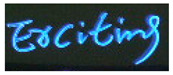	trcitmg	ercitmg	ercitmg	erciting
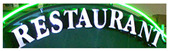	restaurani	restaurani	restaurant	restaurant
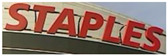	staples	staples	staples	staples
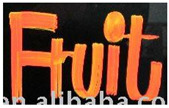	auit	ftuit	ftuit	fruit
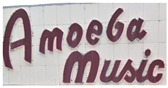	ammbausic	amoecamusic	amoecamusic	amoecamusic
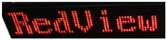	redview	RedView	redview	redview
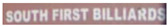	southfirstbilliahds	southfirst billards	south first billiards	south first billiards

## Data Availability

The datasets used in the presented study are openly available at [6,28,35,36].
